# Whole exome sequencing revealed a novel dystrophin-related protein-2 (*DRP2*) deletion in an Iranian family with symptoms of polyneuropathy

**DOI:** 10.22038/ijbms.2019.30754.7414

**Published:** 2019-05

**Authors:** Maryam Tahmasebi-Birgani, Mohammadreza Hajjari, Neda Golchin, Bita Shalbafan, Javad Mohammadi-Asl, Forouzan Sadeghian

**Affiliations:** 1 Department of Medical Genetics, School of Medicine, Ahvaz Jundishapur University of Medical Sciences, Ahvaz, Iran; 2 Department of Genetics, Faculty of Science, Shahid Chamran University of Ahvaz, Ahvaz, Iran; 3 Noor Genetics Lab. Ahvaz, Iran; 4 Iranian Social Security Organization, Labafinejad Hospital, Tehran, Iran; 5 Aboozar Children’s Hospital, Ahvaz Jundishapur University of Medical Sciences, Ahvaz, Iran

**Keywords:** Charcot-marie tooth disease Dystrophin-related protein - 2 gene (*DRP2*), Genetic heterogeneity, Hereditary sensory, Motor-neuropathy, Whole exome sequencing

## Abstract

**Objective(s):**

Charcot-Marie Tooth disease (CMT) is one of the main inherited causes of motor and sensory neuropathies with variable expressivity and age-of onset. Although more than 70 genes have been identified for CMT, more studies are needed to discover other genes involved in CMT. Introduction of whole exome sequencing (WES) to capture all the exons may help to find these genes.

**Materials and Methods:**

Here, we tried to find the genetic cause of the neuropathy in two Iranian brothers using WES. Blood sample was collected from probands and their family members to extract the genomic DNA. The extracted DNA from one of the affected case was subjected for WES. The variant calls were filtered to reveal the pathogenic variant. Presence of the candidate mutation was confirmed using Sanger sequencing. The pathogenic potential of the variant was examined using in silico software. Using ClustalW multiple alignment, the presence of variant in conserved domain of protein was investigated. The parent and another affected boy were also checked for presence of the variant using PCR-sequencing.

**Results:**

The obtained data presented a novel TTC del mutation in CDS 738 of dystrophin related protein 2 (*DRP2*) gene, which was validated by sequencing. The variant was located in a conserved domain of DRP2 protein and predicted as pathogenic. Two affected boys were hemizygous for the mutation and received the mutation from mother.

**Conclusion:**

Here, we provided the evidence for the contribution of *DRP2* in CMT. Also, the symptoms shed light on molecular aspect of this genetically heterogeneous disease.

## Introduction

Charcot-Marie-Tooth disease (CMT) is one of the main causes of hereditary neuropathies with the prevalence of 1/2500 in the United States (1). It is clinically diagnosed with motor and sensory deficits, symmetric distal amyotrophy in lower limbs and hands, steppage gait, foot deformities including pes cavus, pes planus, pes valgus, distal sensory loss in a stocking/glove distribution and decreased or absent deep tendon reflexes (1). Scoliosis and hip dysplasia are also observed in some cases (2). These features have been summarized in Table 1. Generally, the symptoms are variable and depend on the genetic subtypes. This is also true in case of disease age of onset (3). More than 70 known genes are associated with CMT, putting it in the category of heterogeneous diseases, although most of these genes are involved in molecular pathway of Schwann and axons functions (4). The autosomal dominant inheritance is often observed in the CMT families; however, autosomal recessive, X-linked dominant and X-linked recessive pedigrees have been also reported (5). Duplication of 17p^11.2-12^ - *PMP22*gene- is the most frequent chromosomal aberration among CMT cases and is frequently observed in countries such as Germany, Belgium, UK and USA (5). The genetic alterations attributed to *MPZ*, *Cx32* genes as wells as *PMP22* point mutations are also too prevalent (3). There are still some cases with no alteration in *PMP22* or other identified genes indicating that other genes may probably be involved (6). Due to such heterogeneity, conventional molecular diagnostic tool are only effective in 50-70% of cases (7). Around 80% of Mendelian disease-associate variants are located in exonic regions (8). Whole exome sequencing (WES) is a novel, rapid and cost-effective approach in which all the protein coding regions of the genome can be targeted in one run (9). It promises to be helpful in case of heterogeneous disorders like CMT where the conventional mono-targeted strategies fail to diagnose the disease (4). Of note, whole exome sequencing is able to uncover all the mutation types from point mutations to deletion and duplication (10). Here, by means of WES strategy, we found a novel mutation in dystrophin related protein 2 (*DRP2*) gene in two brothers of a family with symptom of neuropathy similar to CMT.

Recently, Brennan *et al.* (2015) identified a hemizygous c.805C-T transition in *DRP2* gene in a 60-year-old man with an intermediate form of CMT disease (7). Since the mutation causes abnormalities recapitulating the *DRP2* knockout model, it potentially represents a novel genetic cause of CMT. Herein, we report a mutation in *DRP2* gene in a family with neuropathy and provide the evidence of *DRP2* contribution in CMT-like symptoms.

**Figure 1:**
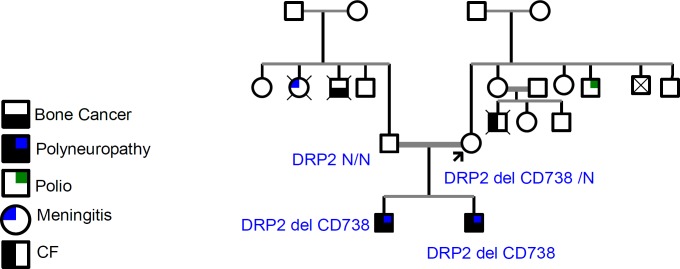
The pedigree of two brothers from a family with consanguinity marriage and symptom of neuropathy

**Figure 2:**
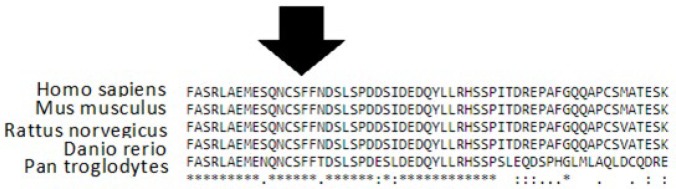
Clustal W multiple alignment revealed that dystrophin related protein-2 (DRP2) deletion has been occurred in a conserved domain of the protein

**Figure 3:**
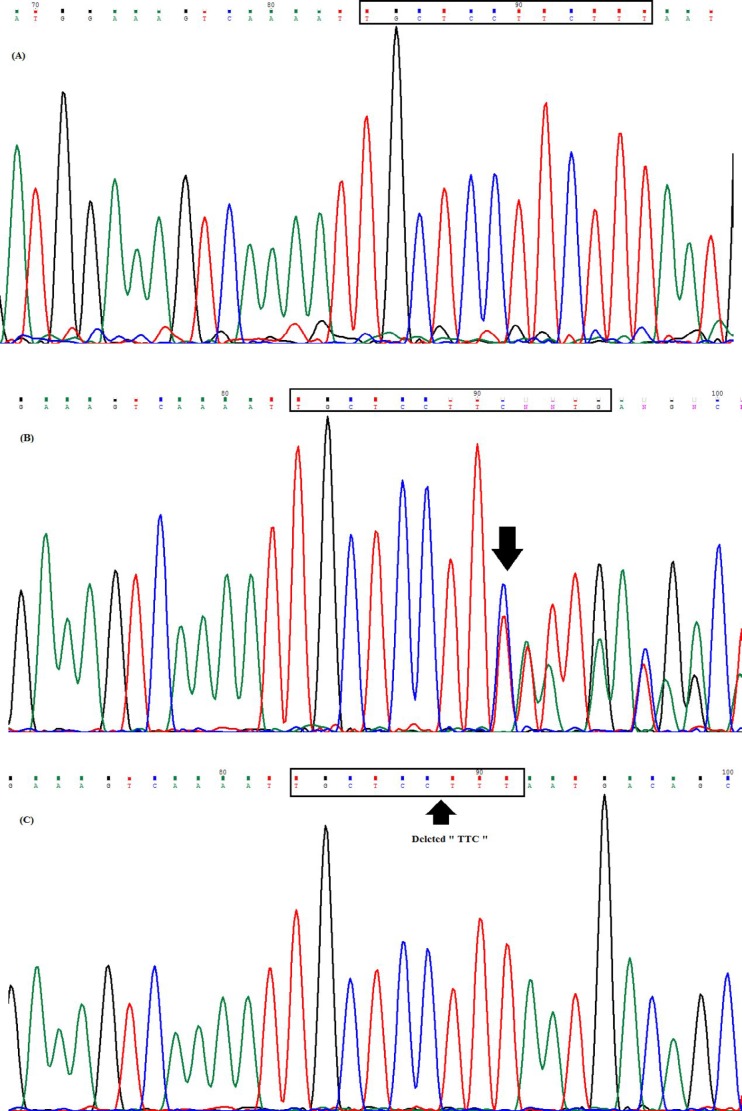
Direct sequencing of the dystrophin related protein-2 (DRP2) gene in an Iranian family suffering from neuropathy; Father (A), Mother (B) and two affected boys (C)

**Table 1:** Sign and symptoms of patients

Sign	**Reference**
Balance difficulty and tremor due to loss of muscle tissue especially in feet	(16)
Muscle fatigue and cramping	(16)
High-arched feet (pes cavus), flat-arched feet (pes planus) or curled toes	(16)
Loss of touch sensation in the feet, ankles, legs, hands, wrists, and arms	(17)
Involuntary grinding of teeth and squinting	(18)
Hearing and vision problems	(18)
Skeletal deformations like Scoliosis	(19)
Neuromuscular hip dysplasia	(20)
Gastrointestinal problems	(18)
Difficulty chewing, swallowing, and speaking due to atrophy of vocal cords	(18)

**Table 2:** Dystrophin related protein-2 (*DRP2*) specific primers

**Primer**	**Sequence**	**PCR product **
***DRP2*** **- Forward primer**	5´- CCAATGATCCTGCTGTGAGA-3´	237 bp
***DRP2*** **- Reverse primer**	5´- GGAGGATATACCCTTCCCAAA-3´	

**Table 3:** Study of the pathogenicity of dystrophin related protein-2 (*DRP2*) deletion. CD738 using *in silico* analysis

**Prediction Server**	**Value**	**Range**
Mutation Taster	Disease causing	Polymorphism to disease causing
PROVEAN	Deleterious	Tolerated to deleterious
FATHMM	Pathogenic	Neutral to Pathogenic

***Case presentation***

The probands were two brothers in a consanguineous family with slowly progressive polyneuropathy and cardiomyopathy. One of the brothers (case 1) was 22 years old. When he was toddler, he could not stand normally. He was clumsy in kneeling and squat position. He had complained ataxic gait since he was fourteen. He referred to pediatric and orthopedic clinics several times without definite diagnosis. At 16 years old, he complained from firm and painless gastrocnemius muscles that removed throughout muscle atrophy, muscle cramps in legs and some abnormal movements like brief myoclonus in his hands. Steppage gait was observed during his physical exam. The flat feet, pes cavus, hammer toes and foot drop in leg suspended position were easily diagnosed. His cardiac functional classified as 1 that means no limitation of physical activity. Ordinary physical activity did not cause undue fatigue, palpitation and dyspnea. Due to both feet drop, inability to toes dorsiflexion in both feet and pes cavus, absence of deep tendon reflexes in 4 limbs, and 4 limbs distal weakness without Babinski sign were observed. Axonal type sensorimotor polyneuropathy was found in both feet and hands during electrophysiological evaluations. No abnormality in Brain MRI was detected. His brother was a 16 years old boy (case 2) with the same problems, although no cardiac abnormality was found in his echocardiography. The micrognathia was found in this case but not in other family members. No abnormality in physical and electrophysiological examination of their mother as a carrier was detected. Flat foot was the only significant finding in their father. No electrophysiological abnormality was detected in his evaluation.

Due to presence of pes cavus, polyneuropathy and ataxia, he was firstly evaluated for Friedrich ataxia. Although cardiomyopathy and low ejection fraction were about 40% and anteroapical hypokinesis was observed, absence of expansion in GAA repeat in *Frataxin* gene ruled it out. Another alternative was Charcot-Maritooth (CMT) disease, which was evaluated using next generation sequencing due to its heterogeneity. 

***Patient recruitment***

The study was ethically approved in Shahid Chamran University of Ahvaz, Iran and all the participant signed the informed consent before joining in this project.

***DNA extraction***

Blood sample (5 ml) was collected from probands and all their available family members in EDTA-containing tubes. 

Genomic DNA was extracted from nucleated cells according to the salting out procedure and was stored at -20 ^°^C before use. The extracted DNAs were checked on 1.5% agarose gel electrophoresis. Additionally, concentration and purity of the genomic DNAs were evaluated by Nanodrop ND-1000 spectrophotometer (Nanodrop Technologies, Wilmington, DE, USA) reading at wave length of 230, 260 and 280 nm.

***Whole exome Sequencing***

DNA extracted from case 1 was sent to Macrogene company (South Korea) for whole exome sequencing to reveal if pathogenic variants exist. The Mutation Taster (http://www.mutationtaster.org), PROVEAN (http://provean.jcvi.org/index.php) and FATHMN (http://www.ensembl.info/ecode/fathmm) were applied to predict if the variants are pathogenic. Consequently, the variants were traced through the family members. To reveal if the *DRP2 *mutation occurs in conserved domain of DRP2, protein sequences from different organisms were recruited from the NCBI (https://www.ncbi.nlm.nih.gov/protein/?term=drp2) and checked for multiple alignment. 

***Polymerase chain reaction (PCR)***

To confirm the variants, the polymerase chain reaction (PCR) was designed using the specific primer flanking the variants’ position. A fragment of 237 bp PCR product was amplified using specific primers flanking the variant-containing region (Table 2). The reaction was carried out in total volume of 25 μl containing 1× reaction buffer, 0.25 mM of each dNTP, 2 pmol/l of each primer, 0.4 μg genomic DNA templates and 1.5 U of Taq DNA polymerase. PCR was optimized in thermocycler and comprised of 5 min denaturation at 95 ^°^C for initial denaturation followed by 35 cycles of denaturation; 95 ^°^C for 30 sec, annealing; 60 ^°^C for 30 sec, extension; 72 ^°^C for 30 sec, final extension; 72 ^°^C for 2 min. A sample without DNA template was included in all PCR reactions as negative control. To credit the reaction, the PCR products were then analyzed on 2% agarose gel electrophoresis dyed with 2% ethidium bromide, and product bands were visualized under ultraviolet light.

***Sanger sequencing***

Using the same primers, Sanger sequencing was performed by Big Dye Terminators (Applied Biosystems 3130 Genetic Analyzer; Applied Biosystems, Foster City, CA, USA).

## Results

***A novel pathogenic deletion in DRP2 gene was confirmed in WES-treated proband***

Following the whole exome sequencing on case 1, the hemizygote variant “*DRP2* del CD738” was found in the proband. The variant was a “TTC” deletion on chromosome X from nucleotide 100510203 to 100510205 (GRCH37/hg19). It has not been previously reported in the dbSNP or literatures. To predict the pathogenicity of the discovered variant, *in silico *analysis was performed using Mutation Taster, PROVEAN and FATHMN, and the results of each method suggested that the variant is a disease causing, deleterious and pathogenic, respectively, and DRP2 protein can be functionally affected (Table 3). To verify the WES-extracted deletion in case 1, *DRP2* gene was amplified using the specific primers flanking the target variant. Sequencing of the amplicon confirmed the variant (Figure 1). As illustrated in Figure 2, the variation has been occurred in conserved domain of DRP2 protein. Therefore, it is not surprising to be functionally deleterious. 

***Sanger sequencing revealed that the other affected brother and their mother also carry the mutation***

The inheritance of “*DRP2* del CD738” was traced through the case 1’s family members including parents and his affected brother (Case 2). DNA extraction and PCR-sequencing was performed in a similar manner with case 1. Similarly, the brother was hemizygous for “*DRP2* del CD738” variant. Although the father was normal, mother was a carrier of “*DRP2* del CD738” variant (Figure 3). Segregation analysis was also performed to check the mutation in other members of the family. These data confirms that the mutation has maternal origin and the observed neuropathy in the pedigree followed an X-linked recessive pattern. 

## Discussion

Conventional molecular strategies for diagnosis of CMT are not reliable, at least in case of heterogenic diseases, so introduction of next-generation sequencing into the diagnostic era of medical genetics laboratories promises to be helpful in case of non-syndromic or idiopathic cases (9).

CMT is one of such heterogeneous disorders with 70 known causative genes that provide different phenotypic subclasses and inheritance pattern (3, 4). Application of whole exome sequencing was strongly advised for CMT to reveal which gene should be considered in affected families (9). 

Here, we presented a novel variant on *DRP2* genes in two brothers of a family with symptom of polyneuropathy. The variant was the deletion of 3 nucleotides TTC in the gene. Mother was the carrier of the variation and the two brothers received the disease allele from their mother. This also suggests an X-linked neuropathy. In human, *DRP2 *is located on Xq^22.1^. It compromises 24 exons with the 45 kb length and preferentially express in brain and spinal cord (16). The presence of two spectrin-like repeats in its structure produce binding sites for Dystroglycan, Syntrophins and Dystrobrevins (17). In a complex with Periaxin and Dystroglycan proteins, DRP2 facilitates the formation of cajal bands in myelinating schwan cells. It also mediates the attachment of extracellular matrix proteins to the schwan cell cytoskeleton like myelin (7). Besides, through Syntrophins, DRP2 may indirectly associate with neural voltage-gated Na^+^ channel (18). Although the association of Periaxin mutations was confirmed in autosomal recessive CMT4F neuropathy, *DRP2* null mice showed weak myelin abnormality. Nevertheless, a recent experiment by Brennan (7) showed a nonsense mutation C.805 C>T in *DRP2 *gene in a 60 years old case of neuropathy. In addition to these reports, there is also evidence in which DRP2 variants are observed in some neurological impairment condition. As an example, whole exome sequencing of a six boys of four unrelated families with X-linked agammaglobulinemia and Mohr–Tranebjaerg syndrome revealed a large deletion in all patients, and in one case deletion included the genes *TIMM8A*, *TAF7L*, and *DRP2. *Of note, the cases suffered from neurological impairments and sensorineural deafness (19). By means of whole exome sequencing and in 10 multiplex families with autism spectrum disorders, Toma *et al*. (20) found a novel nonsense mutation in *DRP2* indicating involvement of this gene as novel candidate gene in autism. All the above-mentioned suggest that *DRP2* can be considered as a novel candidate for CMT genetic counseling assays, although more cases need to be considered by this way. 

## Conclusion

Here, we presented a novel mutation in *DRP2* gene in two Iranian brothers with symptoms of CMT using WES analysis. The mutation was a TTC del in CDS 738of *DRP2 *gene, which was located in a conserved domain of the DRP2 protein. *In silico* analysis predicted the mutation as pathogenic. The boys were hemizygous for the mutation and received the mutation from their mother. Altogether, this information highlighted the *DRP2 *gene as a candidate gene for CMT, although further analysis is necessary. 
